# Altered Apical Morphology (Reverse Architecture): Use of Indirect Ultrasonic Technique for Orthograde MTA Placement in Maxillary Premolars

**DOI:** 10.1155/2016/1046405

**Published:** 2016-05-24

**Authors:** Kapoor Sonali, Agrawal Vineet Suresh, Patel Abhishek, Patel Jenish

**Affiliations:** Department of Conservative and Endodontics, M. P. Dental College and Hospital, Vadodara 390011, India

## Abstract

*Aim*. To report the management and orthograde technique of MTA placement in case of reverse architecture maxillary premolars.* Summary*. Two cases of 17-year-old and 21-year-old female patients were referred to endodontic speciality for management of maxillary premolar having reverse architecture with wide immature open apex like a bell mouth. In both the cases, after control of intraradicular infection, it was decided to use MTA for apexification and obturation of canals. Orthograde placement of MTA is a challenging procedure in terms of length control and condensation especially in divergent irregular reverse architecture wide open apex. A novel technique with the help of finger plugger, sterilized paper point, and ultrasonic agitation for 3D compaction of MTA at apical reverse architecture was used. Thickening of the canal wall and complete apical closure were confirmed one year after the treatment.

## 1. Introduction

The treatment of pulpal injury after eruption of teeth, during the period, that is, 3 yrs, for completion of root development and closure of apex, provides significant challenge for endodontist [1, 2]. During this period, if pulpal exposure occurs due to trauma or caries, necrosis of pulp takes place, ceasing dentin formation and arresting root growth. Resultant immature root will have a wide open apex termed as blunderbuss canal [3].

Bell mouth-like open apex has reverse architecture, that is, larger apical diameter and smaller coronal diameter, leading to improper root canal debridement [4]. It leads to difficulty during filling of root canal due to lack of resistance from apical tissues and absence of natural constriction [4]. Wide apical foramen requires large volume of filling material to be condensed apically, which may extrude in periapical region due to lack of apical seat leading to foreign body reactions [5]. Also, the thin root canal walls will make the tooth more prone to fracture [4].

Apexification is defined as “a method of inducing a calcified barrier in a root with an open apex or the continued apical development of an incompletely formed root in teeth with necrotic pulp” [6]. The classical apexification process with calcium hydroxide is associated with some problems, such as long-term treatment and the risk of teeth fracture [7]. Presently, mineral trioxide aggregate (MTA) obturation technique has become a more favourable option in treating necrotized teeth with open apex owing to its good canal sealing property, biocompatibility, and ability to promote pulp and periapical tissue regeneration [8, 9]. Also, reduced treatment time, less chance of tooth fractures, and fewer dental appointments have added to its advantage [9]. However, orthograde placement of MTA is a challenging procedure in terms of length control in reverse architecture open apex cases which require a specialized sensitive placement technique.

Reverse architecture or wide open apex cases mostly reported in literature are in maxillary anterior teeth and rarely in maxillary premolars [7]. The purpose of this report is to present successful management of two rare cases of maxillary premolars with necrotic pulp and open apex with reverse architecture obturated with MTA. Also, the cases illustrate proper MTA placement technique in reverse architecture wide open apex teeth.

## 2. Case Reports

*Case  1*. A 17-year-old, medically free female patient was referred by some general dentist to the Department of Conservative Dentistry and Endodontics after performing emergency root canal opening in upper right first premolar. Patient suffered from severe pain and intraoral swelling in maxillary right first premolar. These symptoms occurred frequently during the last 5-6 years but have never been treated.

**Figure 1:**
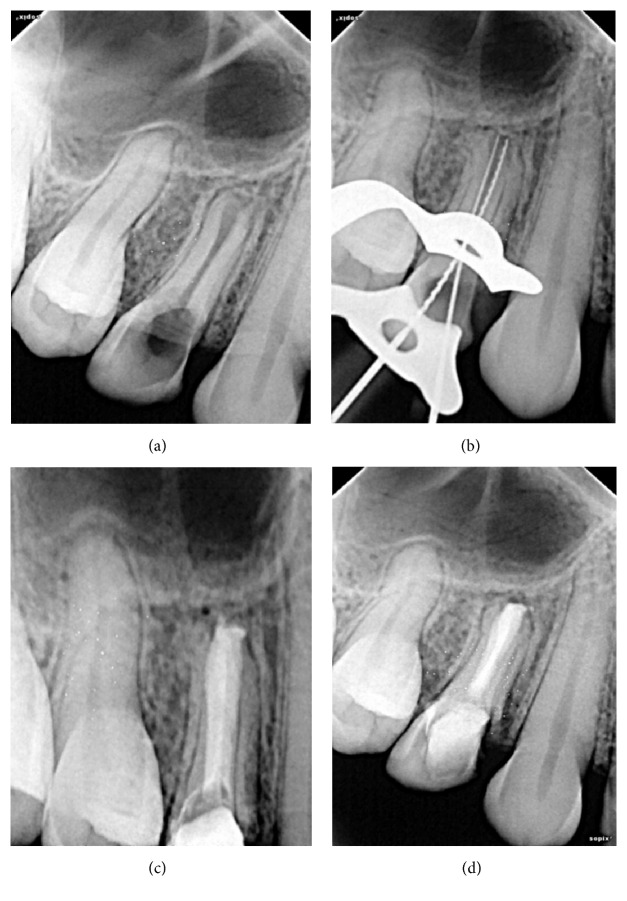
(a) Preoperative radiograph (Case  1). (b) Working length radiograph (Case  1). (c) MTA obturation (Case  1). (d) One-year follow-up radiograph (Case  1).

On clinical examination, there was root canal opening performed in 14 with some carious tooth structure still remaining. No mobility was present but 14 was tender on percussion. Radiographically, tooth appeared to have reverse architecture with wide immature open apex like a bell mouth with thinner root wall and periradicular radiolucency (Figure 1(a)). It was decided that MTA will be used for apexification and complete obturation of both root canals after thorough debridement.

After rubber dam isolation, access cavity was prepared and remaining caries was removed with Endo-Z Bur (Dentsply Maillefer) to enhance the visibility of root canal. An approximate working length was estimated on radiograph for both the buccal and the palatal canals (Figure 1(b)). Both the canals showed lack of resistance in periapical region indicating open apex in both the roots (buccal and palatal). The root canal walls were gently instrumented with stainless steel K-files and irrigated with 5 mL of 2.5% sodium hypochlorite (NaOCl). Canals were dried with paper points. A thick mixture of calcium hydroxide was placed in the canals for 2-week interval and temporary coronal seal was established with CAVIT (3M ESPE, St. Paul, MN, USA).

When patient returned, she was asymptomatic. Calcium hydroxide was removed from the canals using 5 mL of 2.5% NaOCl, and the canals were dried with paper points. A mixture of ProRoot MTA powder (ProRoot MTA, Dentsply Tulsa Dental Specialties, Johnson City, TN, USA) and distilled water was prepared as per manufacturer's instruction. Then, the MTA was placed by orthograde technique in both canals individually as described below.

At first, few mm of MTA was taken on applicator instrument and placed at canal orifice. Using an endodontic finger plugger and back end of sterilized paper point, premeasured to be within 0.5–1 mm of the working length, MTA was packed towards the apex. At this point, indirect ultrasonic agitation (Satelec Acteon, Mérignac, France) of MTA material via plugger was done to create sort of a wet sand flow effect and again it was condensed using back end of sterilized paper point ensuring 3D compaction of MTA at bell mouth-like open apex. Radiograph was taken to confirm correct location of the first pack of MTA apically. After confirmation of proper position, the rest of the canal was filled with MTA incrementally as well as condensing with plugger and paper point. A moist cotton pellet was placed above this condensed MTA for setting and access cavity was temporized with CAVIT (3M ESPE, St. Paul, MN, USA).

At the next appointment, after verifying set of MTA, the tooth was permanently restored with the dentin bonded composite resin (Filtek Z350 XT, 3M ESPE, St. Paul, MN, USA) (Figure 1(c)). The clinical follow-up at 1 year revealed adequate clinical function without clinical symptoms, such as percussion pain, palpation pain, or swelling. Follow-up radiograph at 6 months (Figure 1(d)) also revealed healing of periradicular region.

*Case  2*. A 21-year-old, medically free female patient was again referred by some general dentist to the Department of Conservative Dentistry and Endodontics after performing emergency root canal opening in upper left second premolar. Patient suffered from severe pain in maxillary left second premolar. Patient complained of similar pain 8-9 years back giving history of chewing something hard on the same side with pain subsiding on its own.

**Figure 2:**
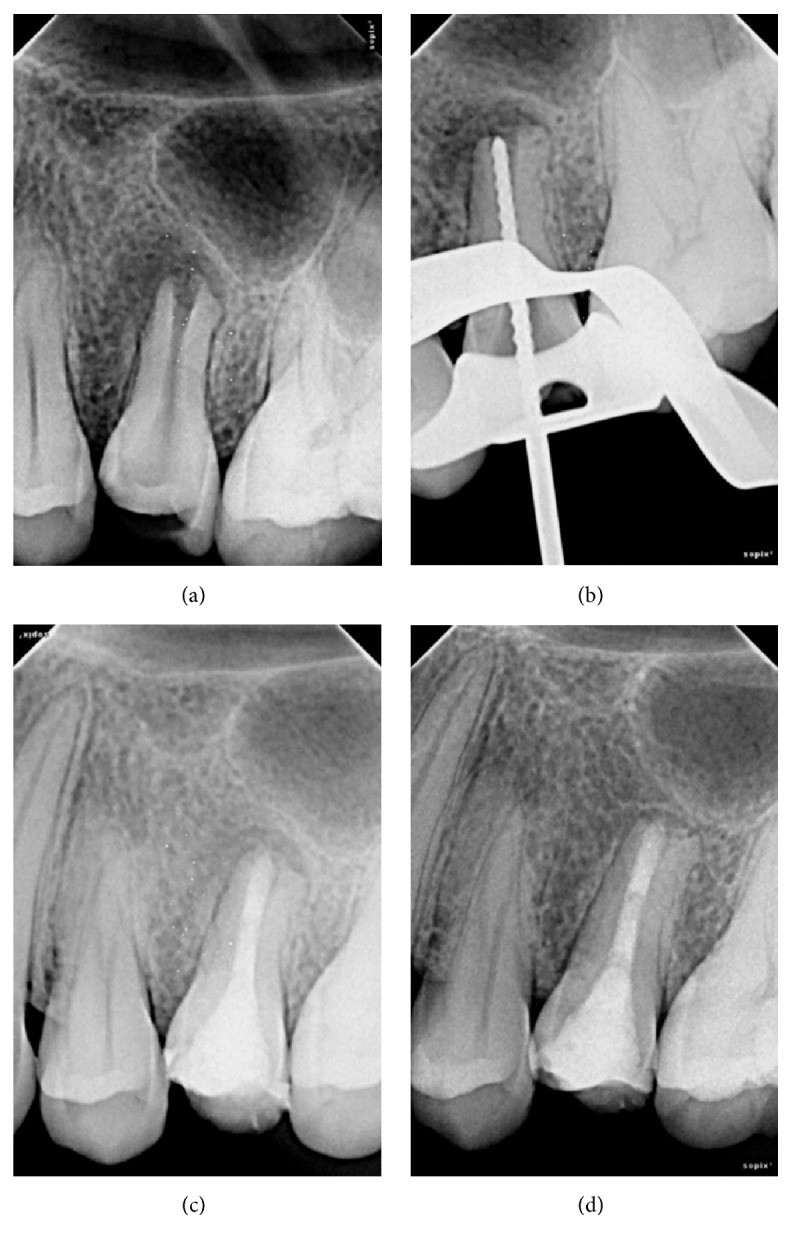
(a) Preoperative radiograph (Case  2). (b) Working length radiograph (Case  2). (c) MTA obturation (Case  2). (d) One-year follow-up radiograph (Case  2).

On clinical examination, there was root canal opening performed in 25 and no mobility was present. Radiographically, tooth appeared to have reverse architecture with wide immature open apex like a bell mouth with thinner root wall and periradicular radiolucency (Figure 2(a)). It was decided that MTA will be used for apexification and complete obturation of the root canal after thorough debridement.

Following the same protocol in Case  1, after rubber dam isolation, access cavity was prepared, working length radiograph was taken (only 1 canal was present in this case) (Figure 2(b)), minimal instrumentation with K-files was done, irrigation with 2.5% NaOCl was performed, and calcium hydroxide dressing was given for 2 weeks. After 2 weeks, calcium hydroxide was removed and MTA was placed in orthograde manner following similar technique described in Case  1. After setting of MTA, permanent restoration was done with composite restoration (Filtek Z350 XT, 3M ESPE, St. Paul, MN, USA) (Figure 2(c)).

Follow-up at 1 year revealed no clinical symptoms and radiograph (Figure 2(d)) shows complete healing of the periapical lesion with regenerated bone and periodontal ligament-like space.

## 3. Discussion

Successful healing of odontogenic tissues, from both a periodontal and endodontic aspect, has developed a quest for such material since last decade. Introduction of MTA has led to this solution as it can induce healing of periapical tissues, such as periodontal ligament, bone, and cementum. MTA has led to thicker and less porous dentin bridge formation and less pulp inflammation compared with calcium hydroxide [7, 10]. MTA induces recruitment and proliferation of undifferentiated cells to form a dentin bridge while reducing inflammation compared with calcium hydroxide [7]. Also, MTA when placed in direct contact with the human dental pulp cells (DPCs) differentiated them into odontoblast-like cells [11]. MTA has also been shown to permit cementoblast attachment and growth as well as the production of mineralized matrix gene and protein expression [12].* In vitro* experiments have shown that MTA upregulated the expression of type I collagen and osteocalcin in osteoblasts after 24 h [12]. Other research studies have shown that MTA stimulates the proliferation of cementoblasts, fibroblasts, and osteoblasts [10–12].

From a clinical point of view, in cases with immature apex with necrotic pulp and inflamed periapical lesion, there is always the presence of tissue fluid or exudation [5]. MTA has unique advantage that is able to set in the presence of moisture. When treating nonvital teeth, main issue is eliminating bacteria from the root canal system. In the above cases, in order to limit bacterial infection before obturation with MTA, irrigation with sodium hypochlorite was done and short-term intracanal calcium hydroxide medication was placed within the canal for two weeks. The rationale was, as instrumentation should be minimal due to thin dentinal walls, complete debridement depends on irrigation and intracanal medicament.

In immature teeth with reverse architecture, the absence of apical constriction complicates the proper placement of MTA within root canal confinements. In addition, the inherent irregularities and divergent nature of the tooth anatomy may affect its adaptation to the dentin walls, predisposing the material to marginal gaps at the dentin interface. In the above cases, access cavities were modified to have enhanced visibility and straight access to root. Orthograde method of MTA placement is more technique sensitive than retrograde method but Hachmeister et al. [13] found that sealing ability of MTA is superior when using orthogradely as apical plug. Aminoshariae et al. [14] reported that hand condensation resulted in better adaptation and fewer voids. Hence, in the above cases, hand condensation technique along with indirect ultrasonic agitation was used for MTA condensation to obtain proper seal and flow of MTA in area of reverse architecture. Various instruments can be used for this precision of MTA placement such as paper points, ultrasonic agitation of the material (creating sort of a wet sand flow effect), and larger hand-files that have been flattened at the tip. Indirect ultrasonic activation means touching the ultrasonic tip with any instrument such as plugger and transforming the vibrating energy to MTA leading to its proper condensation in bell mouth-like area. A study by Yeung et al. [15], comparing the fill density of MTA by hand and ultrasonic compaction, showed that ultrasonication produced a denser MTA fill. A recent study by Parashos et al. [16] concluded that the use of ultrasonics with MTA was useful in improving flow and compaction of MTA, but excessive ultrasonication can adversely impact MTA properties. A suggested time of 2 seconds of ultrasonication per increment presented the best compromise between microhardness values, dye penetration depths, and lack of radiographic voids [16].

MTA has a profound advantage when used as a canal obturation material because of its superior physicochemical and bioactive properties. A study by Hatibović-Kofman et al. [17], on fracture resistance and histological findings of immature teeth treated with mineral trioxide aggregate, showed that the teeth with root treatment with MTA showed the highest fracture resistance at 1 year. As in the above cases, there were thinner root dentin walls remaining, and hence to reinforce the tooth entire canal was obturated with MTA. It has an added advantage of speed of completion of therapy and periapical healing that follows.

Both clinical and radiological examination in follow-up showed healing of periapical lesion and hard tissue formation in apical area of affected teeth. Hence, MTA can be considered very effective in management of immature permanent teeth with open apices and reverse architecture if placed with proper condensation technique. We cannot confirmatively say that regeneration, revascularization, repair, or apexification has taken place in our cases due to the limitation of clinical cases as we cannot take the histological sections to know which type of tissue and cells has led to healing.
